# Classification of Depression and Its Severity Based on Multiple Audio Features Using a Graphical Convolutional Neural Network

**DOI:** 10.3390/ijerph20021588

**Published:** 2023-01-15

**Authors:** Momoko Ishimaru, Yoshifumi Okada, Ryunosuke Uchiyama, Ryo Horiguchi, Itsuki Toyoshima

**Affiliations:** 1 Division of Information and Electronic Engineering, Muroran Institute of Technology, 27-1, Mizumoto-cho, Muroran 050-8585, Hokkaido, Japan; 2 College of Information and Systems, Muroran Institute of Technology, 27-1, Mizumoto-cho, Muroran 050-8585, Hokkaido, Japan

**Keywords:** audio feature, depression, classification model, correlation, graph convolutional neural network

## Abstract

Audio features are physical features that reflect single or complex coordinated movements in the vocal organs. Hence, in speech-based automatic depression classification, it is critical to consider the relationship among audio features. Here, we propose a deep learning-based classification model for discriminating depression and its severity using correlation among audio features. This model represents the correlation between audio features as graph structures and learns speech characteristics using a graph convolutional neural network. We conducted classification experiments in which the same subjects were allowed to be included in both the training and test data (Setting 1) and the subjects in the training and test data were completely separated (Setting 2). The results showed that the classification accuracy in Setting 1 significantly outperformed existing state-of-the-art methods, whereas that in Setting 2, which has not been presented in existing studies, was much lower than in Setting 1. We conclude that the proposed model is an effective tool for discriminating recurring patients and their severities, but it is difficult to detect new depressed patients. For practical application of the model, depression-specific speech regions appearing locally rather than the entire speech of depressed patients should be detected and assigned the appropriate class labels.

## 1. Introduction

Depression is a mood disorder that causes not only psychological symptoms such as loss of pleasure and interest, poor concentration, and hopelessness about the future, but also physical symptoms such as disrupted sleep, eating disorders, and fatigue. According to the World Health Organization [1], there are approximately 280 million people with depression in the world, and the number of patients has been increasing every year [2]. The diagnosis of depression is based on subjective judgments made by physicians through observation and interviews, but quantitative and objective diagnostic methods have not yet been established. Therefore, approximately 50% of actual depressed patients are initially diagnosed as not depressed [3,4]. Such patients cannot receive prompt treatment, resulting in an exacerbation of symptoms and prolonged treatment.

Movement disorders are common in depressed patients because of abnormalities in the basal ganglia of the brain [5]. Naturally, such movement disorders also affect the movements of the vocal organ. Several studies have been published on the speech characteristics of depressed patients. For example, Silva et al. [6] revealed that these movement disorders were related to depression-specific speech features in the voices of depressed patients. In their study, it was shown that multiple audio features extracted from vowels were associated with the clinical state of depression. Scherer et al. [7] found statistically significant differences in audio features related to the degree of tension in speech between depressed patients and nondepressed subjects. Thus, recent studies have revealed that there are audio features peculiar to the speech of depressed patients.

Against this background, speech-based automatic depression classification has been widely studied in recent years to support physicians’ decision-making in the diagnosis of depression. In 2017, a workshop with a subchallenge for depression classification was held [8], and many methods for depression classifications based on deep learning algorithms have been proposed [9,10,11,12,13]. For example, Ma et al. [9] applied a convolutional neural network (CNN) to examine a sound spectrogram and showed that depression could be identified with an accuracy of approximately 50% to 70%. Rejaibi et al. [13] proposed a method based on long short-term memory (LSTM) and showed that depression could be identified with an accuracy of 76%.

Many other speech-based depression classification methods have been proposed, but they all used a single audio feature [9,10,11,12] or multiple audio features independently [13]. In contrast, Airas et al. [14] showed the existence of audio features that were associated with each other. Audio features are physical features that reflect the results of single or complex coordinated movements of various parts in the vocal organs. Hence, in speech-based depression classification, it is critical to consider the relationship among audio features. However, no depression classification method based on the relationship among audio features has been proposed to date.

In this study, we propose a depression classification model for detecting depression and its severity, which can learn speech characteristics based on the correlation among the audio features. The proposed model is constructed using a graph convolutional neural network (GCNN). GCNN is a deep learning model that extends CNN and has been successful in the field of image classification, to apply to graph-structured data [15,16]. The proposed model represents a set of audio features showing strong correlations with each audio feature as a graph structure and performs feature extraction from speech using the respective generated graphs. The three main contributions of this study are as follows: (1) utilizing correlations among audio features for speech-based depression classification; (2) demonstrating better classification performance than existing state-of-the-art methods in the same experimental setting as those existing methods; and (3) presenting a critical issue for future research on automatic depression classification through an experimental setting from a practical perspective that has not been conducted in the existing studies.

The remainder of this study is organized as follows. Section 2 describes the construction method of the proposed model. Section 3 describes the experimental methodologies, and Section 4 presents the experimental results. Section 5 discusses the observations. Finally, Section 6 concludes the study and makes suggestions for potential future work.

## 2. Materials and Methods

### 2.1. Dataset

In this study, we used the DAIC-WOZ dataset [17], which was designed to support the diagnosis of psychological distress states, such as anxiety, depression, and post-traumatic stress. This dataset consists of facial expression data, speech data, text data, and audio feature data during interview responses from 189 subjects (102 males and 87 females). We only used the audio feature data to construct the GCNN-based classification model. The audio feature data is a set of 74-dimensional feature vectors generated from the voiced sound samples taken every 10 ms from the speech data of the DAIC-WOZ dataset.

### 2.2. Definition of Severity Classes of Depression

In the DAIC-WOZ dataset, each subject was assigned a score based on the eight-item Patient Health Questionnaire depression scale (PHQ-8) [18], which is a scale developed to diagnose depression and classify its severity. The PHQ-8 score is calculated as the sum of the subject’s response to eight questions. The eight questions of the PHQ-8 and the levels of depressive severity based on the PHQ-8 score are shown in Table 1 and Table 2, respectively. Although the PHQ-8 score takes integer values from 0 to 24, we defined four severity classes (Nondepression, First stage, Intermediate stage, and Final stage) ranging from 0 to 20 (Table 2), which is based on the literature [10]. Based on the four classes defined here, we conducted the following two experiments: a binary classification of nondepression and depression, and a four-class classification based on severity.

### 2.3. Creation of Feature Vectors for This Study

We excluded three subjects with PHQ-8 scores of 21 or higher following the literature [10] and also one subject with incorrect class labels from the 189 subjects. Thus, we used the remaining 185 subjects’ audio feature data (101 males and 84 females). Here, nine audio features were excluded from the feature vectors because the variance of the values of these audio features was so close to zero, and the correlation coefficients could not be calculated correctly when the similarity graphs in the following section were generated. The remaining 65 audio features were normalized so that the median was 0 and the quartile range was 1. The final audio features used in this study are listed in Table 3. We generated 6,639,782 65-dimensional feature vectors using the methods described above.

### 2.4. Generation of Similarity Graphs

**Figure 1:**
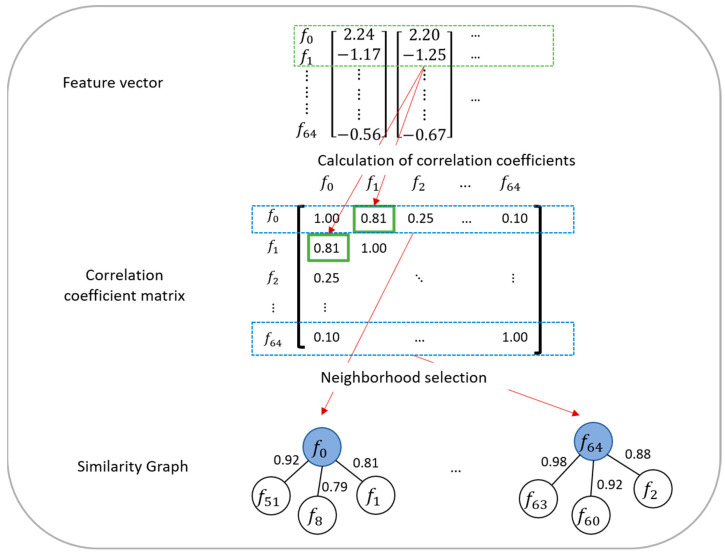
Schematic of similarity graph generation.

In this section, we explain how to generate similarity graphs that are used to account for correlations among audio features in the GCNN model construction. A schematic of the generation of similarity graphs is shown in Figure 1. First, the similarity $S_{x,y}$ between audio features x and y is calculated as follows:

(1) $$S_{x,y}=\left|\frac{\frac{1}{n}\sum_{i=1}^{n}\left(x_{i}-\bar{x}\right)\left(y_{i}-\bar{y}\right)}{\sqrt{\frac{1}{n}\sum_{i=1}^{n}{\left(x_{i}-\bar{x}\right)}^{2}\;}\times \sqrt{\frac{1}{n}\sum_{i=1}^{n}{\left(y_{i}-\bar{y}\right)}^{2}\;}}\right|,$$

Here, n is the total number of feature vectors used as the training data, and $x_{i}$ and $y_{i}$ are the values of x and y in the i-th feature vector, respectively. $\bar{x}$ and $\bar{y}$ are the mean value of x and y across n feature vectors, respectively. Furthermore, m audio features (referred to as neighborhoods) were selected in descending order of similarity for each audio feature. In this study, m was set to 9. Each audio feature and its neighborhoods were represented by nodes and were connected (note that the neighborhoods were not connected). Thus, 65 similarity graphs for 65 audio features were generated.

Defferrard et al. [15] reported that in GCNN models, the similarity-based neighborhood selection was superior to random neighborhood selection in classification accuracy. This is because the random neighborhood selection loses the data structure regarding the association among the neighborhood. Moreover, convolution over all audio features without using neighborhoods is nothing but feature extraction from the entire audio feature vector; hence the similarity relation among audio features cannot be considered. In contrast, by using neighborhood, it is possible to focus on local similarities around each audio feature.

### 2.5. Model Construction and Classification Process

The proposed GCNN model uses the feature vectors and similarity graphs created in Section 2.3 and Section 2.4. This section first describes CNN, which is the basis of GCNN. A brief overview of GCNN is then given, followed by an explanation of the construction of our GCNN model and the classification method.

#### 2.5.1. CNN

CNN is a deep-learning algorithm that has achieved great success in image classification [19]. CNN extracts features from images using convolution operation in the convolutional layer. The convolution operation generates feature maps by sliding a filter matrix over the input image and multiplying and adding the matrices. Typically, the convolution layer is repeated multiple times. The feature map extracted from the final convolutional layer is converted to a vector and input to the fully-connected layer for classification. One of the advantages of CNN is its high translation invariance, which is the ability to identify specific objects in an image even if their positions change.

#### 2.5.2. GCNN

The input to CNN is assumed to be grid-structured data, such as image data, with meaningful pixel order and arrangement. This is because, in the convolutional layer, a square filter matrix is typically used to perform local convolution operations among neighboring pixels. However, GCNN allows convolution operations to be performed on nonadjacent elements based on similarity relationships. GCNN transform convolved elements into graph-structured data based on similarity relationships. In this study, each element, i.e., each audio feature, was represented by a node, and a similarity relationship between two nodes was represented by an edge, as described in Section 2.4. Various GCNNs with different feature similarity calculation methods have been proposed so far [20,21,22]. In this study, we used the GCNN algorithm that uses the absolute value of the correlation coefficient between audio features, as described in Section 2.4.

#### 2.5.3. Training and Classification by GCNN

**Figure 2:**
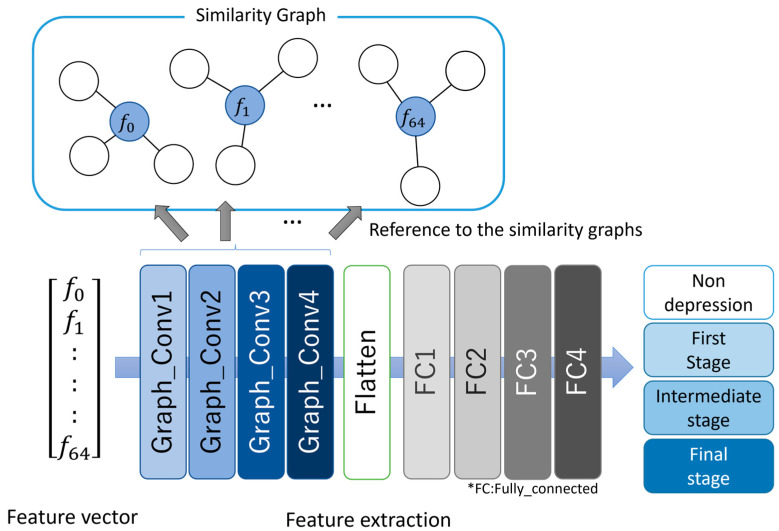
Architecture of the proposed GCNN model.

The proposed model was constructed using the correlation-based GCNN presented in the literature [16]. The architecture of the model and its details are shown in Figure 2 and Table 4, respectively. The input to the GCNN model during the training process was the 65-dimensional feature vectors generated in Section 2.3. First, the convolution operations were performed in the graph convolutional layer on each audio feature and its neighborhoods by referencing the similarity graph. This process is equivalent to performing the convolution operation on image data using a filter in CNN. The input feature vector was converted to a flattened vector and inputted to the subsequent four fully-connected layers via the four graph convolutional layers. Finally, the softmax function was used to convert a vector output from the last fully-connected layer to a probability vector. The cross-entropy error between the probability vector and a one-hot vector corresponding to the true class label was calculated during the training process, Adam [23] was used as an optimization function, and the dropout rate was set to 0.1 in all layers. The learning rate and batch size were set to 0.001 and 1024, respectively. The weights and biases were updated using the backpropagation algorithm.

The 65-dimensional feature vector generated from the subject’s speech was inputted into the model during the classification process, and the output from the model was the class label with the highest probability value.

## 3. Experiments

### 3.1. Experimental Settings

We evaluated the classification performance using a binary classification test between the nondepression and depression classes, as well as a four-class classification test based on the severity. These experiments were conducted under the following two settings.

- Setting 1: Speaker-dependent test

The feature vectors of each class were randomly split into training data (80%), test data (10%), and validation data (10%). In this setting, feature vectors derived from the same subject are included in both the training and test data. This setting is known as speaker-dependent in the field of speaker recognition [24], and it is the same experimental setting as the existing studies [9,10,11,12,13].

- Setting 2: Speaker-independent test

In Setting 1, feature vectors derived from the same subject were included in both the training and test data. However, in actual clinical practice, the patient’s audio data is rarely present in the training data. Therefore, in Setting 2, we divided the subjects in each class into five blocks so that the same subjects would not be assigned to multiple classes, and we performed five-fold cross-validation; that is, subjects for training and testing were completely separated. This setting is known as speaker-independent in the field of speaker recognition [24], and it was an experimental setting that had not been validated in existing studies on speech-based depression classification. Setting 2 allows us to evaluate the classification performance from a more practical point of view.

### 3.2. Evaluation Indices

The classification performance was evaluated using the following indices:

(2) $$\mathrm{Precision}=\frac{TP}{TP+FP}\;,$$

(3) $$\mathrm{Recall}=\frac{TP}{TP+FN}\;,$$

(4) $$F1-\mathrm{score}=\frac{2\mathrm{Recall}\cdot \mathrm{Precision}}{\mathrm{Recall}+\mathrm{Precision}}.$$

Here, TP, TN, FP, and FN indicate the number of true positives, true negatives, false positives, and false negatives, respectively.

## 4. Results

### 4.1. Classification Results for Setting 1

#### 4.1.1. Results for Binary Classification

Table 5 shows the confusion matrix obtained in the binary classification experiment between the nondepression and depression classes. The scores for each index calculated from the classification results are shown in Table 6, which shows that the binary classification was achieved with high accuracy.

#### 4.1.2. Results of Four-Class Classification

Table 7 shows the confusion matrix obtained from the four-class classification experiment. The scores for each index calculated from the classification results are shown in Table 8, which shows that the four-class classification was achieved with high accuracy. However, the accuracies of the other depression classes (First stage, Intermediate stage, and Final stage) were somewhat lower than that of the nondepression class. This is because the class distribution in the training data was imbalanced and the number of feature vectors of the nondepression class was considerably larger than those of the depression classes.

### 4.2. Classification Results for Setting 2

#### 4.2.1. Results for Binary Classification

Table 9 shows an example of the confusion matrix obtained in the binary classification experiment between the nondepression and depression classes. The mean scores for each index obtained from the five-fold cross-validation are shown in Table 10. The classification accuracies for both classes were significantly lower than those in Setting 1. In particular, the depression class showed a considerable decrease in accuracy. This is because many depressed patients were incorrectly classified as nondepressed, as shown in Table 9. These results show that identifying depressed patients who were excluded from the training data was much more difficult.

#### 4.2.2. Results of Four-Class Classification

Table 11 shows an example of the confusion matrix obtained from the four-class classification experiment. The mean scores for each index obtained from the five-fold cross-validation are shown in Table 12. The classification accuracies for all the classes were significantly lower than those in Setting 1. Similar to the results in Section 4.2.1, most prediction results were biased toward the nondepression class. These results show that identifying depressed patients who were not included in the training data was much more difficult.

## 5. Discussion

### 5.1. Discussion on the Results of Setting 1

Table 13 and Table 14 compare classification accuracy in binary classification and four-class classification with existing methods, respectively. All of these existing studies used the same dataset as our study. These tables show that the proposed model outperformed the other methods. Based on these results, the proposed model could appropriately represent and learn individual speech characteristics. However, in all the classification experiments, the classification accuracy of the depression classes (First, Intermediate, and Final stages) was lower than the nondepression class. This is because of the large bias in the number of training data among the classes. The number of feature vectors used as the training data for each class in the binary and four-class classification experiments is shown in Table 15 and Table 16. According to these tables, the number of feature vectors in the nondepression class was considerably larger than in the other classes. Such imbalanced data cause poor classification accuracy for minority classes [25]. Therefore, it is necessary to implement undersampling and data augmentation techniques, as well as to increase the number of training data for these classes to further improve the accuracy of the depressed classes.

Below, we discuss the usefulness of the proposed model through comparison with the existing studies. Srimadhur et al. [10] and Sardari et al. [12] both classifyied depression by applying CNN-based models to raw speech waveforms. As a result, the F1-scores for nondepression and depression were 78.00% and 77.00% in [10], and 71.00% and 70.00% in [12], respectively. Ma et al. [9] and Muzammel et al. [11] classified depression by applying CNN-based models to a Mel frequency spectrogram and a vowels/consonants spectrogram, respectively. As a result, the F1-scores for nondepression and depression were 70.00% and 52.00% in [9], and 90.00% and 77.00% in [11], respectively. The above four studies all provided the models using a single type of audio feature, i.e., speech waveform or spectrogram. On the other hand, Rejaibi et al. [13] attempted depression classification using multiple types of audio features, 60-dimensional MFCC and 53 audio features, similar to our study. Note that their study was conducted under the assumption that each audio feature was independent. In their study, the F1-scores for nondepression and depression were 85.00% and 46.00%, respectively. In contrast, the proposed model enables the learning that focused on the correlation among multiple types of audio features based on GCNN. This is a novel approach that has not been found in the existing studies. We belive that this idea lead to a high classification accuracy of 97.80% for nondepression and 94.64% for depression, which is significantly higher than the existing methods.

### 5.2. Discussion on the Results of Setting 2

In Setting 1, the same subjects were included in both the training and test data. This setting is an experiment to evaluate classification performance on already trained patients, allowing us to estimate the model performance in detecting patients with recurrent depression. However, from a practical standpoint, it is also important to evaluate generalization performance for new as well as trained patients.

Thus, in Setting 2, we completely separated the subjects between the training and test data to evaluate the classification performance of the model from a more practical perspective. To the best of our knowledge, no studies have shown classification results with the same experimental setting as Setting 2. The classification accuracy of Setting 2 was considerably lower than that of Setting 1 (Table 9 and Table 10), indicating that classifying subjects that were not included in the training data was extremely difficult. There are two main reasons for this. The first is that, similar to Setting 1, the number of training data for the nondepression class was significantly greater than those for the depression classes. Imbalanced training data results in misclassification in most classes. Most of the feature vectors for the depression classes were misclassified as nondepression (Table 9 and Table 11). Furthermore, the method used to collect training data (feature vectors) from the speech of depressed patients was inappropriate. In this study, we generated feature vectors in both Settings 1 and 2, assuming that speech features specific to depressed patients appeared in the entire speech. However, the speech features specific to depressed patients are likely to appear locally in the speech [26]. Therefore, it is necessary to assign different class labels to feature vectors obtained from depression-specific speech regions and normal (or nondepressive) speech regions contained in the speech of depressed patients. However, in this study, feature vectors derived from the normal speech regions were treated as depression class data. Note that the existing studies have also generated training data without distinguishing between depression-specific and normal speech regions. To improve the classification accuracy of depressed patients, the different speech regions in depressed patients must be adequately detected and assigned correct class labels. Even in that case, the difference in the number of training data between the nondepression and depression classes must become more pronounced, necessitating the resolution of the data imbalance problem.

### 5.3. Correlation between the Audio Features

**Figure 3:**
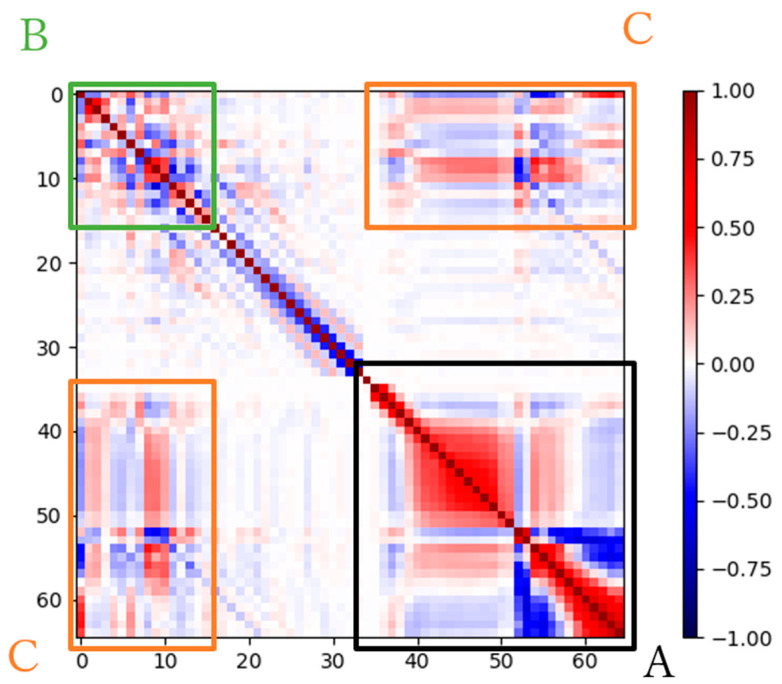
Heat map for the correlation coefficients among the 65 audio features.

The proposed model learns speech characteristics based on correlations among audio features. There are audio features that show relationships with each other, as mentioned in Section 1 [14]. In this section, we discuss the correlations among the 65 audio features used in this study. A heat map showing the correlation coefficients among the 65 audio features is shown in Figure 3. Here, we focus on three areas, A, B, and C, where particularly high correlation coefficients were observed. Region A contained HMPDM and HMPDD, each of which consisted of multidimensional audio features; specifically, it shows that there is a high correlation among different dimensions of the same type of audio features. In region B, high correlations were observed between NAQ and H1H2 and between MDQ and Rd. These correlations were reported in the literature [14,27]. In region C, a high correlation was observed between NAQ and HMPDD. NAQ is a useful biomarker for identifying depression [7]. Therefore, HMPDD, which has a high correlation with NAQ, can be a new biomarker for diagnosing depression. We believe that clarifying the correlations among audio features effective for depression classification using AI-based techniques will be a promising approach in the identification of useful biomarkers for the diagnosis of depression. In the future, we will conduct a more detailed investigation for that purpose.

**Figure 4:**
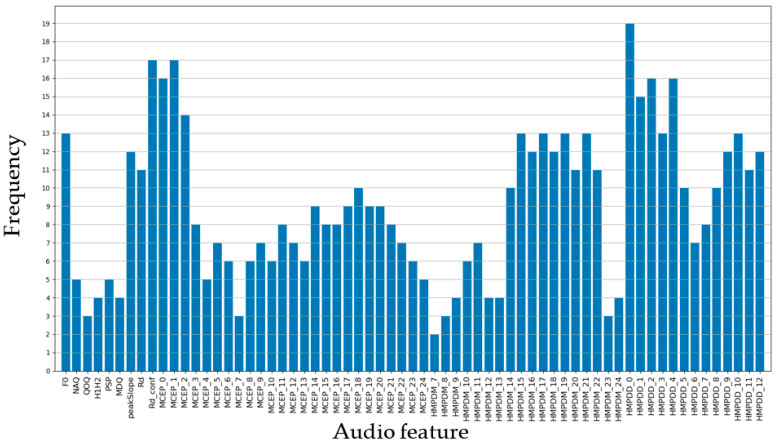
Number of times selected as the neighborhood for each audio feature.

The proposed model performed the convolution operations on each audio feature and its neighborhoods. Therefore, the neighborhoods can have a significant impact on the classification performance of the model. Here, using Figure 4, each audio feature is compared and evaluated based on the number of times it was selected as a neighborhood. As mentioned above, audio features composed of multiple dimensions, such as MCEP, HMPDM, and HMPDD, tend to be selected as neighborhoods more frequently because they show high correlation with each other among the audio features of the same type. On the other hand, although F0, peakSlope, Rd, and Rd_conf are single-dimensional audio features, they were selected as neighborhoods more frequently than others. According to Silva et al. [6] and Scherer et al. [7], F0 and peakSlope were the audio features that showed statistically significant differences between depressed and nondepressed subjects. In addition, Rd and Rd_conf are biomarkers for detecting the strained voice [18] that is often observed in depressed patients [7]. F0, peakSlope, Rd, and Rd_conf are audio features that can be used for depression classification regardless of individual differences, and therefore may have been effective in the classification performance of the model.

Gender difference is an important aspect to be considered in audio-based depression classification. Rejaibi et al. [13] showed that the classification accuracies using the respective models for males and females were better than those of the mixed gender model. This is due to the difference in vocal organs between males and females. However, our model was constructed without distinguishing between the sexes. Similar to the study by Rejaibi et al., the classification accuracy of our model can be affected by gender; hence, the use of models for respective genders should be considered in the future. Airas et al. [14] showed that gender differences existed in strength of correlation among audio features. According to their work, for example, QOQ and Rd showed high correlation in males, but low correlation in females. Considering the gender differences among such audio features is likely to improve the classification accuracy of the model. However, in this study, we used the same neighborhoods for both genders. In order to account for gender differences in audio features, it is necessary to introduce a mechanism to dynamically select effective neighborhoods for each gender.

### 5.4. Discussions on the Practical Application of the Model

Three main issues must be addressed for the practical application of the model. The first is to improve the classification accuracy for new patients not included in the training data. To this end, as discussed in Section 5.2, it is necessary to improve the method of collecting training data from the speech of depressed patients. Furthermore, the number of subjects used for the training data in Setting 2 was 108 for the nondepression class and 40 for the depression class, resulting in extremely imbalanced data among the classes. Data imbalance leads to lower accuracy for minority classes [28]. Therefore, it is necessary to increase the number of depressed subjects and perform data augmentation. Moreover, it may be effective to introduce Focal Loss [29] as a loss function in the model in order to promote the learning of minority classes that are difficult to identify. The second is to improve the proposed model to a noise-robust method. The speech data used in the experiments were recorded in a quiet environment using the same recording equipment for all of the subjects. However, it is not always possible to record speech under such favorable conditions. From a more practical viewpoint, it is important to achieve highly accurate classification even for noisy speech recorded by low-cost devices. For this purpose, a noise reduction process [30] must be introduced in the preprocessing of the speech data. The third is model compression. Generally, neural network models necessitate a large number of computational resources to handle a large number of parameters. Therefore, high-performance computers are required. To realize widespread use in medical practice and personal use, simpler models that can run even on small-scale electronic devices such as smartphones and wearable devices must be developed. For this problem, model compression techniques [31,32] will be an effective approach.

## 6. Conclusions

We conclude this study as follows.

1. In the classification experiment, Setting 1, in which the same subjects were allowed to be included in both the training and test data, the classification accuracy of the proposed model was considerably higher than that of the existing state-of-the-art methods. Hence, the proposed model can be an effective tool for detecting recurrent patients based on their past data, i.e., training data.
2. In the classification experiment, Setting 2, in which the subjects between the training and test data were completely separated, the classification accuracy of the proposed method was considerably lower than that in Setting 1. Hence, it is extremely difficult to classify new patients who are not included in the training data.
3. Speech-based depression classification studies, including ours, have assumed that the entire speech of depressed patients exhibits depression-specific speech features. However, it is likely that speech characteristics peculiar to depressed patients appear locally rather than in the entire speech. Therefore, training data for depressed patients should be generated using only depression-specific speech regions.

We believe that speech-based depression classification will be an effective technology to support objective diagnosis in the medical field. Despite the recent momentum in automatic depression classification research, as reported by Malhotra et al. [33], none of the methods have achieved practical performance. The third point mentioned above is a critical issue that must be resolved in order to improve the ability to detect depression. In the future, we will first address this particular issue, as well as introduce noise reduction and model compression techniques to improve the model performance to a practical level.

## Figures and Tables

**Table 1:** Eight questions of the PHQ-8.

Over the Last 2 Weeks, How Often Have You Been Bothered by the Following Problems?		Not at All	Several Days	More than Half the Days	Nearly Every Day
1	Little interest or pleasure in doing things	0	1	2	3
2	Feeling down, depressed, or hopeless	0	1	2	3
3	Trouble falling or staying asleep, or sleeping too much	0	1	2	3
4	Feeling tired or having little energy	0	1	2	3
5	Poor appetite or overeating	0	1	2	3
6	Feeling bad about yourself—or that you are a failure or have let yourself or your family down	0	1	2	3
7	Trouble concentrating on things, such as reading the newspaper or watching television	0	1	2	3
8	Moving or speaking so slowly that other people could have noticed. Alternatively, being so fidgety or restless that you have been moving around a lot more than usual	0	1	2	3

**Table 2:** Severity levels according to the PHQ-8 score.

PHQ-8 Scores	0	1	2	3	4	5	6	7	8	9	10	11	12	13	14	15	16	17	18	19	20
Diagnostic	Nondepression										Depression										
Severity level	Nondepression										First stage				Intermediate stage			Final stage			

**Table 3:** Audio features used in this study.

Audio Features	Summary
F0	Fundamental frequency
NAQ	Normalized Amplitude Quotient
QOQ	Quasi Open Quotient
H1H2	First two harmonics of the differentiated glottal source spectrum
PSP	Parabolic Spectral Parameter
MDQ	Maxima Dispersion Quotient
PeakSlope	Spectral tilt/slope of wavelet responses
Rd	Shape parameter of the Liljencrants-Fant model of the glottal pulse dynamic
Rd_conf	How well the glottal model fits the signal (0–1)
MCEP_0-24	Mel cepstral coefficient
HMPDM_7-24	Harmonic Model and Phase Distortion Mean
HMPDD_0-12	Harmonic Model and Phase Distortion Deviations

**Table 4:** Details of the structure of the proposed GCNN model.

Layer	Input Data Size	Output Data Size	Number of Kernels	Kernel Size	Dropout	Activation Function
Graph_conv1	65 × 1	65 × 64	64	1 × 9 × 64	False	ReLU
Graph_conv2	65 × 64	65 × 128	128	64 × 9 × 128	False	ReLU
Graph_conv3	65 × 128	65 × 256	256	128 × 9 × 256	False	ReLU
Graph_conv4	65 × 256	65 × 512	512	256 × 9 × 512	False	ReLU
Flatten	65 × 512	33,280	-	-	-	-
Fully_connected1	33,280	4096	-	-	True	ReLU
Fully_connected2	4096	1024	-	-	True	ReLU
Fully_connected3	1024	256	-	-	True	ReLU
Fully_connected4	256	2/4	-	-	True	softmax

**Table 5:** Confusion matrix obtained by the binary classification in Setting 1.

		Predicted Class	
		Nondepression	Depression
True Class	Nondepression	460,129	10,150
	Depression	10,593	183,106

**Table 6:** Classification performance for the binary classification in Setting 1.

	Nondepression	Depression
Precision	97.75%	94.75%
Recall	97.84%	94.53%
F1-score	97.80%	94.64%

**Table 7:** Confusion matrix obtained by the four-class classification in Setting 1.

		Predicted Class			
		Nondepression	First Stage	Intermediate Stage	Final Stage
True Class	Nondepression	462,138	3887	1673	2581
	First stage	4097	102,225	276	442
	Intermediate stage	1762	248	36,411	150
	Final stage	1868	356	97	45,767

**Table 8:** Classification performance for the four-class classification in Setting 1.

	Nondepression	First Stage	Intermediate Stage	Final Stage
Precision	98.36%	95.79%	94.68%	93.52%
Recall	98.27%	95.50%	94.40%	95.17%
F1-score	98.31%	95.65%	94.54%	94.34%

**Table 9:** An example of the confusion matrix obtained by the binary classification in Setting 2.

		Predicted Class	
		Nondepression	Depression
True Class	Nondepression	743,704	189,919
	Depression	249,557	89,865

**Table 10:** Classification performance for the binary classification in Setting 2.

	Nondepression	Depression
Precision	70.87%	29.90%
Recall	75.81%	24.89%
F1-score	73.10%	26.47%

**Table 11:** An example of the confusion matrix obtained by the four-class classification in Setting 2.

		Predicted Class			
		Nondepression	First Stage	Intermediate Stage	Final Stage
True Class	Nondepression	765,562	32,894	12,623	34,815
	First stage	194,094	19,756	7399	2594
	Intermediate stage	36,519	887	1594	686
	Final stage	79,429	5350	1331	1619

**Table 12:** Classification performance for the four-class classification in Setting 2.

	Nondepression	First Stage	Intermediate Stage	Final Stage
Precision	71.31%	25.45%	4.04%	7.71%
Recall	80.79%	17.57%	2.78%	5.20%
F1-score	75.69%	19.67%	3.23%	6.00%

**Table 13:** Comparison of the classification accuracy between our model and the existing methods in the binary classification under Setting 1.

Author	Year	Method	Feature	Precision		Recall		F1-Score	
				Nondepression	Depression	Nondepression	Depression	Nondepression	Depression
Ma et al. [9]	2016	CNN + LSTM	Mel-scale filter bank features	100.00%	35.00%	54.00%	100.00%	70.00%	52.00%
Srimadhur et al. [10]	2020	CNN	The raw speech samples	76.00%	79.00%	80.00%	74.00%	78.00%	77.00%
Muzammel et al. [11]	2020	CNN	Vowel and consonant spaces acoustic features	88.00%	81.00%	92.00%	73.00%	90.00%	77.00%
Sardari et al. [12]	2022	CNN Auto-encoder + Support Vector Machine	Raw audio	70.00%	71.00%	72.00%	68.00%	71.00%	70.00%
Rejaibi et al. [13]	2022	LSTM	MFCC, 53 audio features	78.00%	69.00%	94.00%	35.00%	85.00%	46.00%
Our model	2022	GCNN	65 audio features	97.75%	94.75%	97.84%	94.53%	97.80%	94.64%

**Table 14:** Comparison of the classification accuracy between our model and the existing methods in the four-class classification under Setting 1.

Author	Severity Level	Precision	Recall	F1-Score
Srimadhur et al. [10]	Nondepression	92.00%	65.00%	76.00%
	First stage	62.00%	81.00%	7.00%
	Intermediate stage	34.00%	69.00%	45.00%
	Final stage	8.00%	75.00%	15.00%
Our model	Nondepression	97.97%	98.37%	98.17%
	First stage	95.30%	95.01%	95.16%
	Intermediate stage	95.16%	93.09%	94.11%
	Final stage	94.85%	93.29%	94.06%

**Table 15:** The number of feature vectors used as the training data of each class in the binary classification.

Binary Classification	
Nondepression	4,702,789
Depression	1,936,993

**Table 16:** The number of feature vectors used as the training data of each class in the four-class classification.

Four-Class Classification	
Nondepression	4,702,789
First stage	1,070,398
Intermediate stage	385,713
Final stage	480,882

## Data Availability

Not applicable.

## References

[1] Depression. https://www.who.int/news-room/fact-sheets/detail/depression.

[2] World Health Organization (2017). Depression and Other Common Mental Disorders: Global Health Estimates.

[3] Mitchell A.J., Vaze A., Rao S. (2009). Clinical diagnosis of depression in primary care: A meta-analysis. Lancet.

[4] Katon W., Von Korff M., Lin E., Bush T., Ormel J. (1992). Adequacy and duration of antidepressant treatment in primary care. Med. Care.

[5] Caligiuri M.P., Ellwanger J. (2000). Motor and cognitive aspects of motor retardation in depression. J. Affect. Disord..

[6] Wegina J.S., Leonardo L., Melyssa K.C.G., Anna A.A. (2021). Voice Acoustic Parameters as Predictors of Depression. J. Voice.

[7] Scherer S., Stratou G., Gratch J., Morency L.P. Investigating voice quality as a speaker-independent indicator of depression and PTSD. Proceedings of the INTERSPEECH 2013.

[8] Ringeval F., Schuller B., Valstar M., Gratch J., Cowie R., Scherer S., Mozgai S., Cummins N., Schmitt M., Pantic M. Avec 2017: Real-life depression, and affect recognition workshop and challenge. Proceedings of the 7th Annual Workshop on Audio/Visual Emotion Challenge.

[9] Ma X., Yang H., Chen Q., Huang D., Wang Y. Depaudionet: An efficient deep model for audio based depression classification. Proceedings of the 6th International Workshop on Audio/Visual Emotion Challenge.

[10] Srimadhur N.S., Lalitha S. (2020). An end-to-end model for detection and assessment of depression levels using speech. Procedia Comput. Sci..

[11] Muzammel M., Salam H., Hoffmann Y., Chetouani M., Othmani A. (2020). AudVowelConsNet: A phoneme-level based deep CNN architecture for clinical depression diagnosis. Mach. Learn. Appl..

[12] Sardari S., Nakisa B., Rastgoo M.N., Eklund P. (2022). Audio based depression detection using Convolutional Autoencoder. Expert Syst. Appl..

[13] Rejaibi E., Komaty A., Meriaudeau F., Agrebi S., Othmani A. (2022). MFCC-based recurrent neural network for automatic clinical depression recognition and assessment from speech. Biomed. Signal Process. Control..

[14] Airas M., Alku P. Comparison of multiple voice source parameters in different phonation types. Proceedings of the INTERSPEECH 2007.

[15] Defferrard M., Bresson X., Vandergheynst P. Convolutional neural networks on graphs with fast localized spectral filtering. Proceedings of the Advances in Neural Information Processing Systems 29.

[16] Hechtlinger Y., Chakravarti P., Qin J. (2017). A generalization of convolutional neural networks to graph-structured data. arXiv.

[17] Gratch J., Artstein R., Lucas G., Stratou G., Scherer S., Nazarian A., Wood R., Boberg J., DeVault D., Marsella S. The distress analysis interview corpus of human and computer interviews. Proceedings of the Ninth International Conference on Language Resources and Evaluation.

[18] Kroenke K., Strine T.W., Spitzer R.L., Williams J.B., Berry J.T., Mokdad A.H. (2009). The PHQ-8 as a measure of current depression in the general population. J. Affect. Disord..

[19] Krizhevsky A., Sutskever I., Hinton G.E. (2017). Imagenet classification with deep convolutional neural networks. Commun. ACM.

[20] Roux N., Bengio Y., Lamblin P., Joliveau M., Kégl B. Learning the 2-D Topology of Images. Proceedings of the Advances in Neural Information Processing Systems 20.

[21] Belkin M., Niyogi P. Laplacian eigenmaps and spectral techniques for embedding and clustering. Proceedings of the Advances in Neural Information Processing Systems 14.

[22] Henaff M., Bruna J., LeCun Y. (2015). Deep convolutional networks on graph-structured data. arXiv.

[23] Kingma D.P., Ba J. (2014). Adam: A method for stochastic optimization. arXiv.

[24] Schuller B., Müller R., Lang M., Rigoll G. Speaker independent emotion recognition by early fusion of acoustic and linguistic features within ensemble. Proceedings of the INTERSPEECH 2005-Proceeding European Conference on Speech Communication and Technology.

[25] Yan Y., Chen M., Shyu M.L., Chen S.C. Deep learning for imbalanced multimedia data classification. Proceedings of the 2015 IEEE International Symposium on Multimedia (ISM).

[26] Salekin A., Eberle J.W., Glenn J.J., Teachman B.A., Stankovic J.A. (2018). A weakly supervised learning framework for detecting social anxiety and depression. Proc. ACM Interact. Mob. Wearable Ubiquitous Technol..

[27] Gobl C., Yanushevskaya I., Chasaide A.N. The relationship between voice source parameters and the Maxima Dispersion Quotient (MDQ). Proceedings of the INTERSPEECH 2015.

[28] Haixiang G., Yijing L., Shang J., Mingyun G., Yuanyue H., Bing G. (2017). Learning from class-imbalanced data: Review of methods and applications. Expert Syst. Appl..

[29] Lin T.Y., Goyal P., Girshick R., He K., Dollár P. (2020). Focal loss for dense object detection. ITPAMI.

[30] Kantamaneni S., Charles A., Babu T.R. (2022). Speech enhancement with noise estimation and filtration using deep learning models. Theor. Comput. Sci..

[31] Peng P., You M., Xu W., Li J. (2021). Fully integer-based quantization for mobile convolutional neural network inference. Neurocomputing.

[32] Choudhary T., Mishra V., Goswami A., Sarangapani J. (2022). Inference-aware convolutional neural network pruning. Future Gener. Comput. Syst..

[33] Malhotra A., Jindal R. (2022). Deep learning techniques for suicide and depression detection from online social media: A scoping review. Appl. Soft Comput..

